# Characterization of the dielectric properties of water and methanol in the D-band using a quasi-optical spectroscopy

**DOI:** 10.1038/s41598-019-55126-6

**Published:** 2019-12-06

**Authors:** Xiaoming Liu, Junsheng Yu

**Affiliations:** 1 School of Physics and Electronic Information, Anhui Normal University, Wuhu Anhui, 241002 China; 2 Anhui Provincial Engineering Laboratory on Information Fusion and Control of Intelligent Robot, Wuhu Anhui, 241002 China; 3 School of Electronic Engineering and Computer Science, Queen Mary University of London, London, E1 4NS UK; 4 School of Electronic Engineering, Beijing University of Posts and Telecommunications, Beijing, 100876 China

**Keywords:** Electrical and electronic engineering, Electronic properties and materials

## Abstract

This work presents the measurement of the permittivities of water and methanol in the D-band. Water is a reference medium for dielectric measurement. The dielectric permittivity of water in the millimeter wave range is a fundamental parameter in many applications, and needs to be investigated systematically. The measurement is conducted using a quasi-optical spectroscopy, which is an improved free-space method more suitable for the millimeter wave range. The theoretical formulae are derived using the signal-flow chart method, which is developed specially for multi-layer operation. This model enables one measure liquid samples. A non-calibration method has been developed to retrieve the permittivity. Water and methanol are measured at several temperatures. The measured results agree with published results in a 4% discrepancy. This work will add new measured data to the permittivities of water and methanol over the whole D-band.

## Introduction

Millimeter wave (mm wave) technology has seen increasing applications in a wide range of areas, such as radio astronomy, microwave remote sensing and radar detection^1–3^. In design of mm wave components, the permittivity is a key parameter that determines the RF performance. For instance, the real part of the permittivity determines the central frequency and the imaginary part (also the loss tangent) determines the insertion loss and gain of a mm wave device^4,5^. In addition, the permittivity is also a basic parameter in the study of electromagnetic wave with matters^6^. In this connection, determination of the permittivity in the mm wave range is a fundamental work deserves as much effort as possible.

Water is a reference medium in many cases where a calibration/benchmark measurement is required^7,8^. Consequently, prior knowledge of the permittivity of water is a necessity before a precise measurement can be conducted. Unfortunately, due to its high polar nature, water exhibits apparent relaxation beyond the microwave range, showing a strong frequency-dependent property^9^. Particularly in the mm wave range, the permittivity of water varies significantly with the frequency. Though many results of water permittivity have been published in the literature, a reliable method is still to be established for broadband measurement in the mm wave range. Ellison has built a dielectric model (hereafter referred to as Ellison model) of water based on data collected from many research groups^8^. The Ellison model is based on broadband fitting of the collected data. Unfortunately, data in the D-band is not sufficiently abundant. Only three discrete frequencies have been reported (see Appendix B in ref. ^8^). Later on, an updated model was presented in ref. ^10^. However, the measured data in the D-band is still relative few. Liebe also established a dielectric model for water below 1 THz^9^. Unfortunately, there is no data in the D-band. In Downing’s work^11^, the investigated frequency range is 300 GHz – 150 THz. Ray presented a broadband complex refractive model for ice and water^12^. The measured data, however have a gap between 75GHz–200 GHz. In Rosenkranz’s work^13^, only two frequencies, 150 GHz and 170 GHz in the D-band were examined. In view of these facts, this work is to measure the dielectric property of water in the D-band, aiming at providing more complete data, so that a more reliable model may be developed.

There are several methods of dielectric measurement for liquid samples, such as open-ended coaxial line (OCL) method, transmission line (TL) method, and free space (FS) method. The OCL method is a simple way to measure the permittivity of liquid samples. However, it requires a reference medium, and often causes inaccurate value in the mm wave range^14,15^. The drawback of the TL method is that the dimension of any TL decrease with the increase in frequency, since it is comparable to the wavelength^16^. For instance, the rectangular waveguide in the D-band is 1.651 mm by 0.826 mm according to the EIA-WR standard. Such size is too small to conduct reliable measurement. Also, measurement was done using dispersive Fourier transform spectroscopy (DFTS)^17^ and terahertz time domain spectroscopy (THz-TDS)^18^. The DFTS technique is more suitable for far-infrared or infrared region. The reliable region for a THz-TDS is 0.5 THz – 2.5 THz, which is shown in Fig. 4 of ref. ^18^. The FS method is a broadband technique providing low-loss and multi-polarization transmission. Being based on electronic technique, both the phase and amplitude of transmission/reflection coefficients can be obtained.

This paper presents a systematic FS method for liquid sample measurement in the D-band. The FS spectroscopy is based on a quasi-optical (QO) system, which provides a good beam quality. In order to verify the flow-chart method and the QO system, as well as characterize the permittivity of water, measurement was conducted on de-ionized water (DI-water) and methanol of 99% purity. Methanol serves as a second sample for system verification, considering that it is easily available in laboratory. It is demonstrated that such method can be used in other frequency bands and applied to other liquid samples.

## Methods

### Quasi-optical spectroscopy

**Figure 1:**
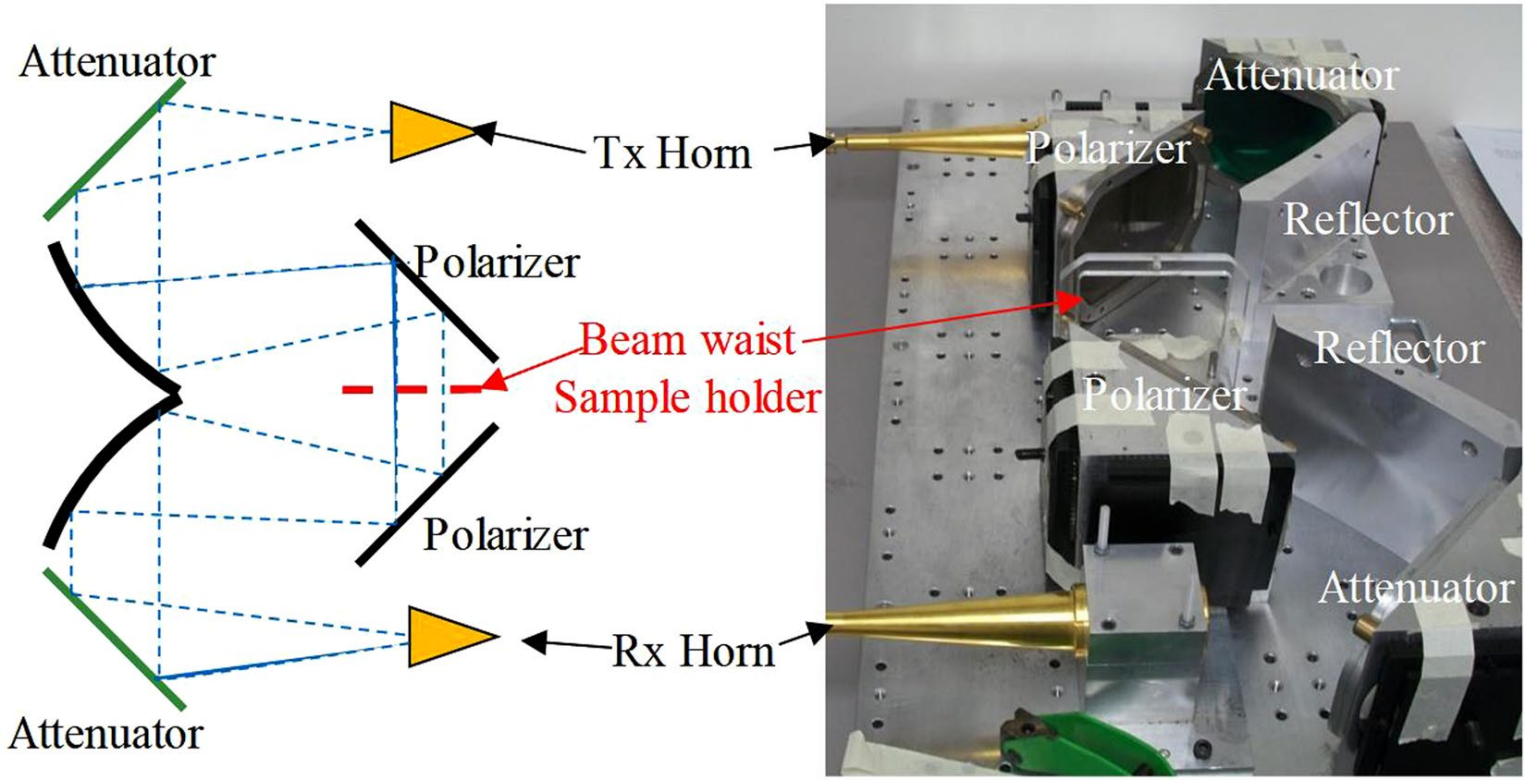
The quasi-optical spectroscopy for dielectric property characterization in the millimeter wave range.

There are many FS measurement systems for dielectric measurement^19,20^. Quasi-optical based spectroscopy is more preferred due to the following reasons. One is that it provides very broadband operation; another is that the beam can be controlled efficiently, leading to very low transmission loss; and the third one is that the polarization can be manipulated with good precision^21^. The QO spectroscopy is shown in Fig. 1. The system was fabricated in Thomas Keating Ltd, UK. It consists two identical corrugated horns, two ellipsoidal reflectors, two polarizers and two attenuators. A customized sample holder is placed at the location of the beam waist, where the beam has a planar wave front.

The corrugated horns are used for transmission (Tx Horn) and receiving (Rx Horn), respectively. The ellipsoidal reflectors are used to refocus the divergent beam. The polarizers are fabricated to eliminate cross-polarization in this system. And the attenuators are occasionally used for biological samples in case of damaging these samples. For a normal sample, the attenuators are replaced with two planar metallic reflectors.

**Figure 2:**
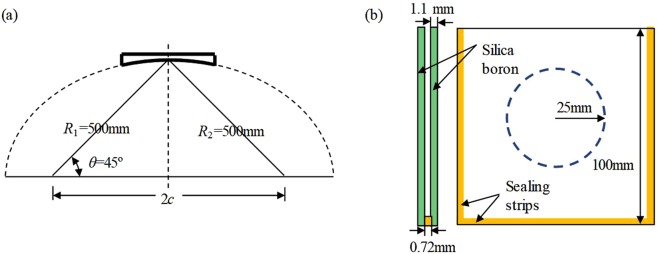
Schematic of (**a**) the ellipsoidal mirrors, and (**b**) the sample holder for liquid samples.

Corrugated horns are extensively investigated, the design routines have been summarized in ref. ^22^. Interested readers are also referred to ref. ^23^ for more theoretical description. Good linear polarization can be produced by using these corrugated horns. The ellipsoidal mirrors are illustrated in Fig. 2a, where *R*_1_ and *R*_2_ are the distance from the foci to the optical center, and *c* is the half focal length of the ellipsoidal mirror. In this system, one has *R*_1_ = *R*_2_ = 500 mm, and θ = 45°. By the QO theory^21^, the equivalent focal length of such reflector can be written as

1 $$F={R}_{1}{R}_{2}/({R}_{1}+{R}_{2}).$$

Since *R*_1_ = *R*_2_ = 500 mm, *F* finds to be 250 mm. The polarizer is a wire grid made of tungsten lines^24^. The sample holder is fabricated from silica boron (*Instrument Glasses Ltd*) with three sides sealed and the top side left open, as shown in Fig. 2b. Such a sample holder keeps a planar structure. Injection of liquid sample can be easily done using a syringe. The thickness of silica boron is 1.1 mm, and the length is 100 mm. Since the beam waist is 25 mm, the edge taper is less than −30dB, which meets the requirements on a QO system. The gap between the two plates of silica boron is 0.72 mm. The provided dielectric constant of the silica boron is 4.6–0.017j at 1 MHz. It has to be mentioned that the feeds are fixed at the equivalent focal points so that broadband operation can be realized^21^. In this work, we restrict the measurement in the D-band.

**Figure 3:**
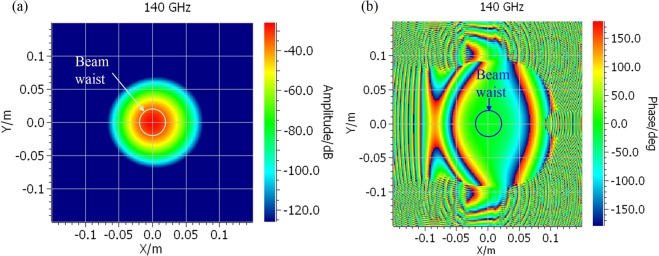
Field distribution of system at the sample holder. (**a**) Amplitude; (**b**) Phase.

To characterize the beam quality at the location of the sample holder, a physical optical (PO) program has been used for field calculation. The predicted field distribution is plotted in Fig. 3. It is shown that the amplitude is of good symmetry. The phase in the area of the beam waist is uniform, showing a good approximation of plane wave.

### Theoretical analysis of multi-layer structure using transfer matrix

It is seen that for liquid sample, the sample holder is a multi-layer structure. Since the sample is placed at the beam waist, the electromagnetic waves are almost planar wave that the propagation can be analyzed using the signal-flow graph method. The signal-flow graph method has been discussed in many textbooks, such as ref. ^25^.

**Figure 4:**
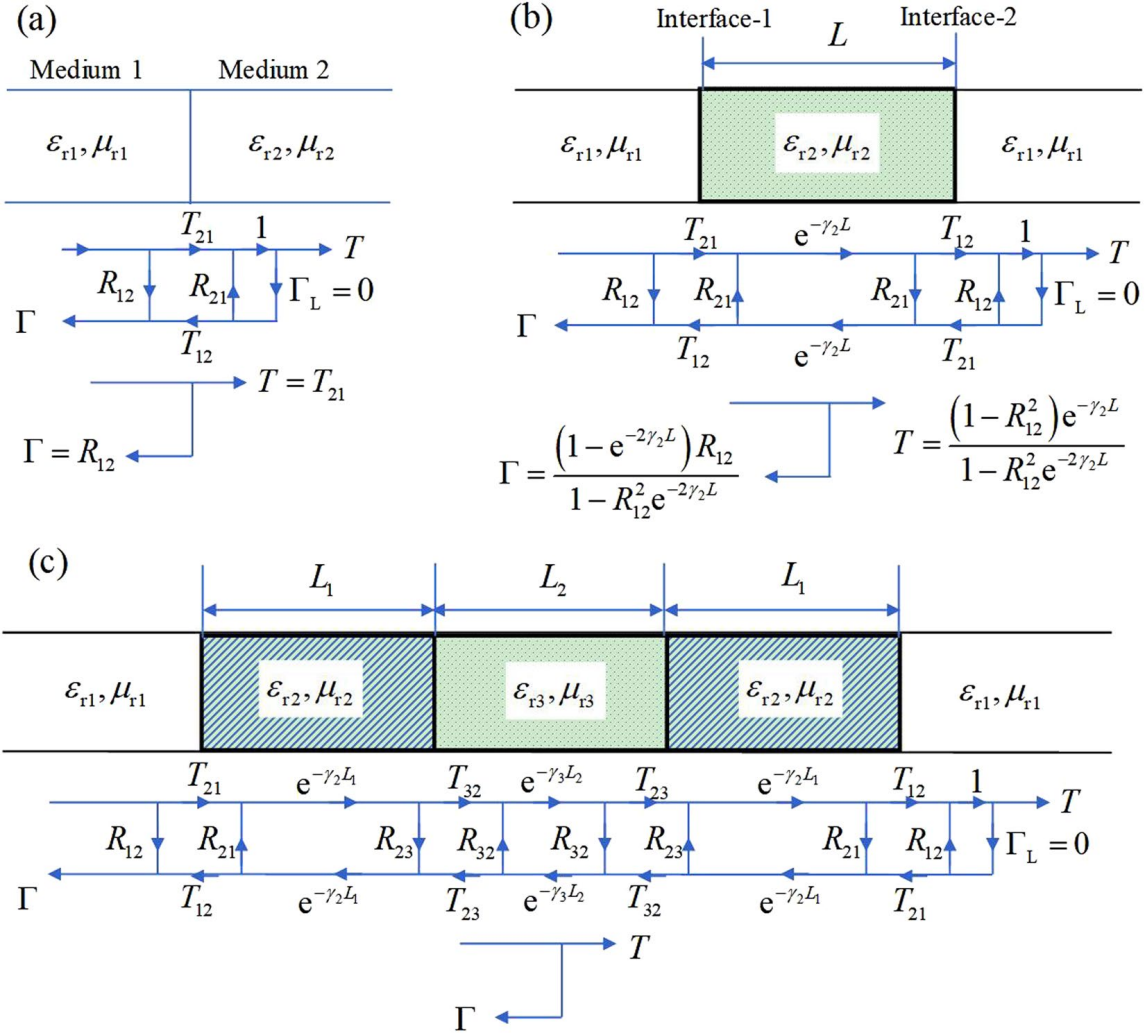
Multi-layer structure analysis using the signal-flow graph method. (**a**) Interface; (**b**) single layer; (**c**) three layers.

In Fig. 4a, an interface is formed between medium 1 and medium 2. Using the signal-flow graph method one has the reflection and transmission coefficients as

2 $$\{\begin{array}{c}\Gamma ={R}_{12}=\frac{\sqrt{{\varepsilon }_{{\rm{r1}}}{\mu }_{{\rm{r2}}}}-\sqrt{{\varepsilon }_{{\rm{r2}}}{\mu }_{{\rm{r1}}}}}{\sqrt{{\varepsilon }_{{\rm{r1}}}{\mu }_{{\rm{r2}}}}+\sqrt{{\varepsilon }_{{\rm{r2}}}{\mu }_{{\rm{r1}}}}}\\ T={T}_{21}=\frac{2\sqrt{{\varepsilon }_{{\rm{r1}}}{\mu }_{{\rm{r2}}}}}{\sqrt{{\varepsilon }_{{\rm{r1}}}{\mu }_{{\rm{r2}}}}+\sqrt{{\varepsilon }_{{\rm{r2}}}{\mu }_{{\rm{r1}}}}}\end{array}.$$

For a non-magnetic material, it can be reduced to

3 $$\{\begin{array}{c}\Gamma ={R}_{12}=\frac{\sqrt{{\varepsilon }_{{\rm{r}}1}}-\sqrt{{\varepsilon }_{{\rm{r}}2}}}{\sqrt{{\varepsilon }_{{\rm{r}}1}}+\sqrt{{\varepsilon }_{{\rm{r}}2}}}\\ T={T}_{21}=\frac{2\sqrt{{\varepsilon }_{{\rm{r}}1}}}{\sqrt{{\varepsilon }_{{\rm{r}}1}}+\sqrt{{\varepsilon }_{{\rm{r}}2}}}\end{array},$$

which is in line with the results based on boundary conditions.

In Fig. 4b, a slab of sample is placed in air, creating two interfaces. By applying the model of Fig. 4a and equating the sample with a transmission line of length *L*, the resultant reflection and transmission coefficients are

4 $$\{\begin{array}{c}\Gamma =\frac{(1-{{\rm{e}}}^{-2{\gamma }_{2}L}){R}_{12}}{1-{R}_{12}^{2}{{\rm{e}}}^{-2{\gamma }_{2}L}}\\ T=\frac{(1-{R}_{12}^{2}){{\rm{e}}}^{-{\gamma }_{2}L}}{1-{R}_{12}^{2}{{\rm{e}}}^{-2{\gamma }_{2}L}}\end{array},$$

where *R*_12_ is shown in Eq. (3) and *γ*_2_ is

5 $${\gamma }_{2}=\frac{{\rm{j2}}\pi f\sqrt{{\varepsilon }_{{\rm{r2}}}}}{{\rm{c}}}.$$

Equation (4) is equivalent to the analysis using wave equation. In this connection, the methods of signal-flow graph can be applied to multi-layer structure (Fig. 4c). The final expressions for the reflection and transmission coefficients are too long to present in this context. Interested readers to these simplification steps are directed to the supplementary document. Alternatively, one may resort to symbolic computation software, such as *Mathematica* and *Maple*. By using iterative procedures, the calculation will be significantly simplified.

To this end, the theoretical expressions for the reflection and transmission coefficients have been derived. Particularly, a theoretical method has been established for analysis of the multi-layer structure. The next step is to find a proper retrieval method from the measured data. In a general case, the Vector Network Analyzer (VNA) is used to measure the reflection and transmission coefficients, denoted as the S-parameter. There are four parameters, i.e. *s*_11_ for the reflection of port 1, *s*_21_ for the transmission from port 1 to port 2, *s*_12_ for the transmission from port 2 to port 1 and *s*_22_ for the reflection of port 2. These parameters have to be measured based on rigorous calibration procedures in a conventional two-port network. For free-space measurement, calibration can be conducted using through-reflection-line (TRL) or through-reflection-Matching (TRM) method. The TRL method is not best for the QO system since the movement of any components would introduce beam distortion. Also, the TRM method requires high-quality matching load. Actually, only the calibration of the waveguide ports is needed. For reflection measurement, a polished metallic plate can be used as reference measurement. And for transmission measurement, air serves as background. By subtracting the measured signal of background from that of the sample, the reflection/transmission coefficients of the sample can be obtained. Such method would significantly reduce the complexity of calibration.

An analytic solution from the measured data to the permittivity is almost impossible, while numerical method is more efficient due to greatly enhanced computation capability. It is seen that reflection and transmission coefficients are functions of the permittivity. Set error functions as

6 $$\{\begin{array}{c}F({\varepsilon ^{\prime} }_{{\rm{r}}},\,{\varepsilon ^{\prime\prime} }_{{\rm{r}}})={s}_{21}-T\\ G({\varepsilon ^{\prime} }_{{\rm{r}}},\,{\varepsilon ^{\prime\prime} }_{{\rm{r}}})={s}_{11}-\Gamma \end{array}.$$

For FS method, the transmission coefficients can be readily obtained with reference measurement on air. Calibration of the reflection is more complicated. In consideration of this, the first error function in Eq. (6) is used in this work. Therefore, it can be written as

7 $$F({\varepsilon ^{\prime} }_{{\rm{r}}},{\varepsilon ^{\prime\prime} }_{{\rm{r}}})=f({\varepsilon ^{\prime} }_{{\rm{r}}},{\varepsilon ^{\prime\prime} }_{{\rm{r}}})+{\rm{j}}g({\varepsilon ^{\prime} }_{{\rm{r}}},\,{\varepsilon ^{\prime\prime} }_{{\rm{r}}}).$$

Its Jacobian matrix finds to be

8 $$J=|\begin{array}{cc}\frac{\partial f({\varepsilon ^{\prime} }_{{\rm{r}}},{\varepsilon ^{\prime\prime} }_{{\rm{r}}})}{\partial {\varepsilon ^{\prime} }_{{\rm{r}}}} & \frac{\partial f({\varepsilon ^{\prime} }_{{\rm{r}}},{\varepsilon ^{\prime\prime} }_{{\rm{r}}})}{\partial {\varepsilon ^{\prime\prime} }_{{\rm{r}}}}\\ \frac{\partial g({\varepsilon ^{\prime} }_{{\rm{r}}},{\varepsilon ^{\prime\prime} }_{{\rm{r}}})}{\partial {\varepsilon ^{\prime} }_{{\rm{r}}}} & \frac{\partial g({\varepsilon ^{\prime} }_{{\rm{r}}},{\varepsilon ^{\prime\prime} }_{{\rm{r}}})}{\partial {\varepsilon ^{\prime\prime} }_{{\rm{r}}}}\end{array}|.$$

**Figure 5:**
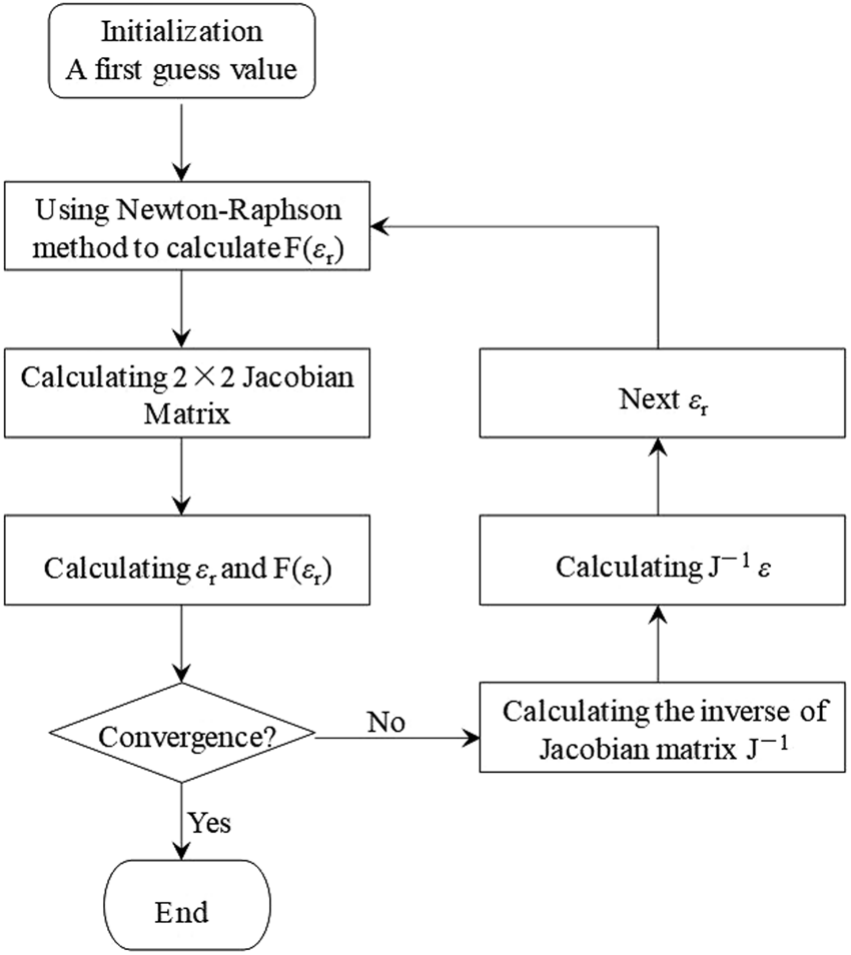
The flow chart of Newton-Raphson method solving for permittivity.

There are many ways for solving the solution of a function. The Newton-Raphson method^26^ was employed in this work. Through a few rounds of iteration, the permittivity can be retrieved. The flowchart of the iteration procedure using the Newton-Raphson method to solve for permittivity is illustrated in Fig. 5.

## Results

Measurements were conducted using a VNA (Agilent N5230C) at several temperatures 18 °C, 26 °C, and 33 °C. The measurement steps are: (a) system warming up for 1 hour; (b) measure air as background; (c) measure the single layer silica boron glass to determine its permittivity in the D-band; (d) measure the samples. Measurement of air serves as a background so as to obtain the phase of the sample.

**Figure 6:**
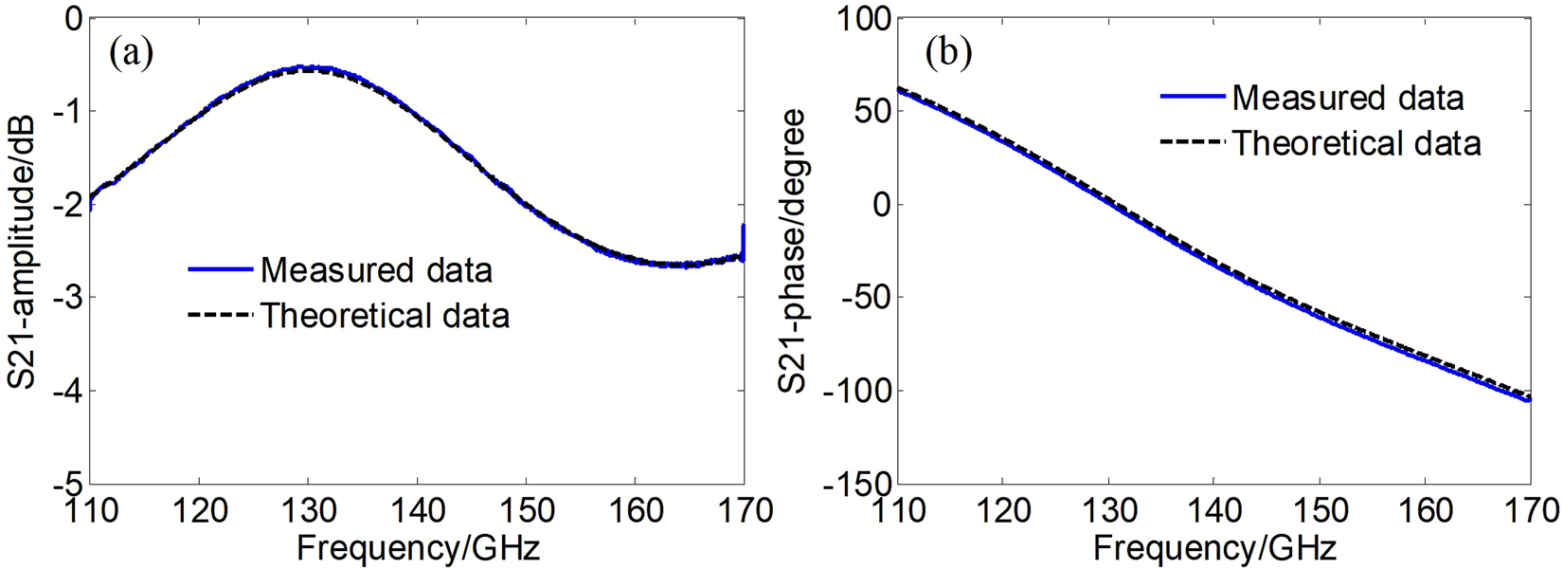
Comparison of the *s*_21_ between measurement and theoretical results of silica boron glass. (**a**) Amplitude; (**b**) phase.

**Figure 7:**
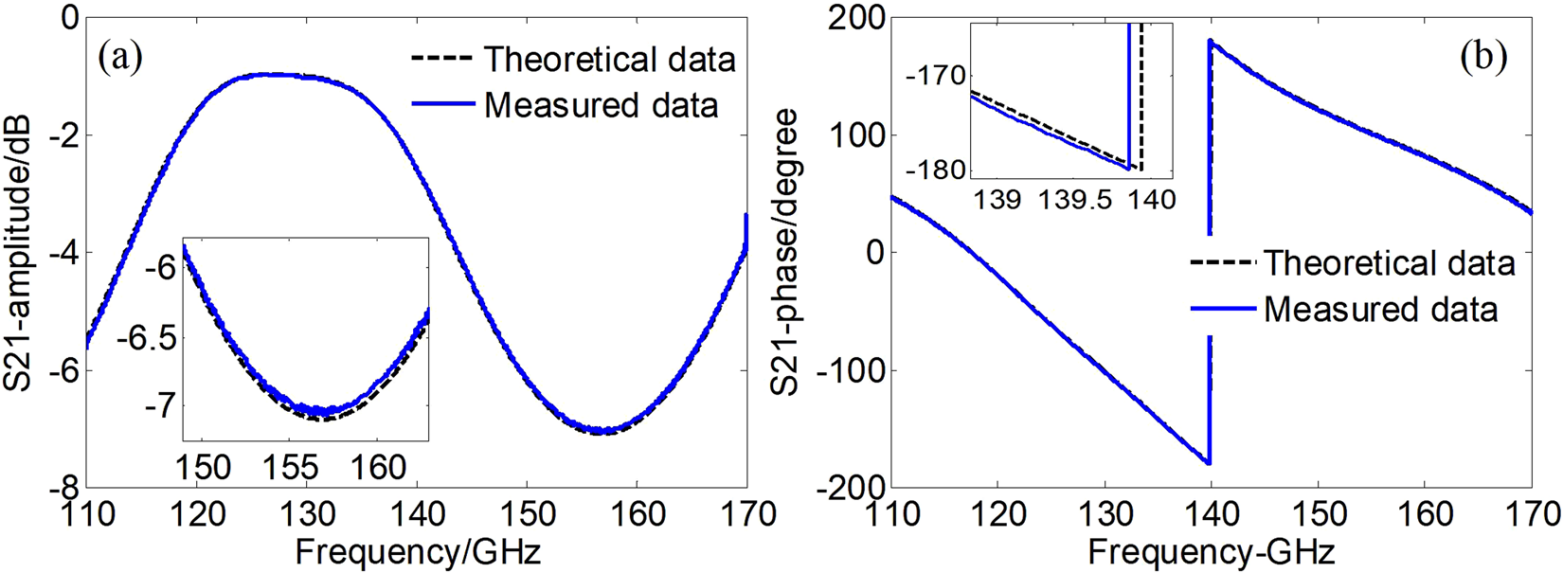
Comparison of the *s*_21_ between measurement and theoretical results of the sample holder. (**a**) Amplitude; (**b**) phase.

By applying the Newton-Raphson method and doing a linear fitting, the extracted permittivity of the silica boron glass is 4.35–j0.066 at 110 GHz and linearly changed to 4.32–j0.082 at 170 GHz. To check the convergence of the calculation, these values were put back to the theoretical formula. It is found from Fig. 6 that the agreement between calculation and measurement is very well. The amplitude discrepancy is within 0.1 dB and the phase deviation is within 3°. Such a precision is sufficient for liquid sample measurement. It can be seen that even for solid sample, the dielectric constant is changing with the frequency. The loss factor increases with the frequency in the D-band. The sample holder was also measured, with the comparison presented in Fig. 7. The retrieved dielectric permittivity of the silica boron was feed into the theoretical model to calculate the *s*_21_. This step is very important since it is an approach to checking the quality of the sample holder, for instance, the width of the gap. Fortunately, the agreement between the calculation and the measurement is sufficiently good.

**Figure 8:**
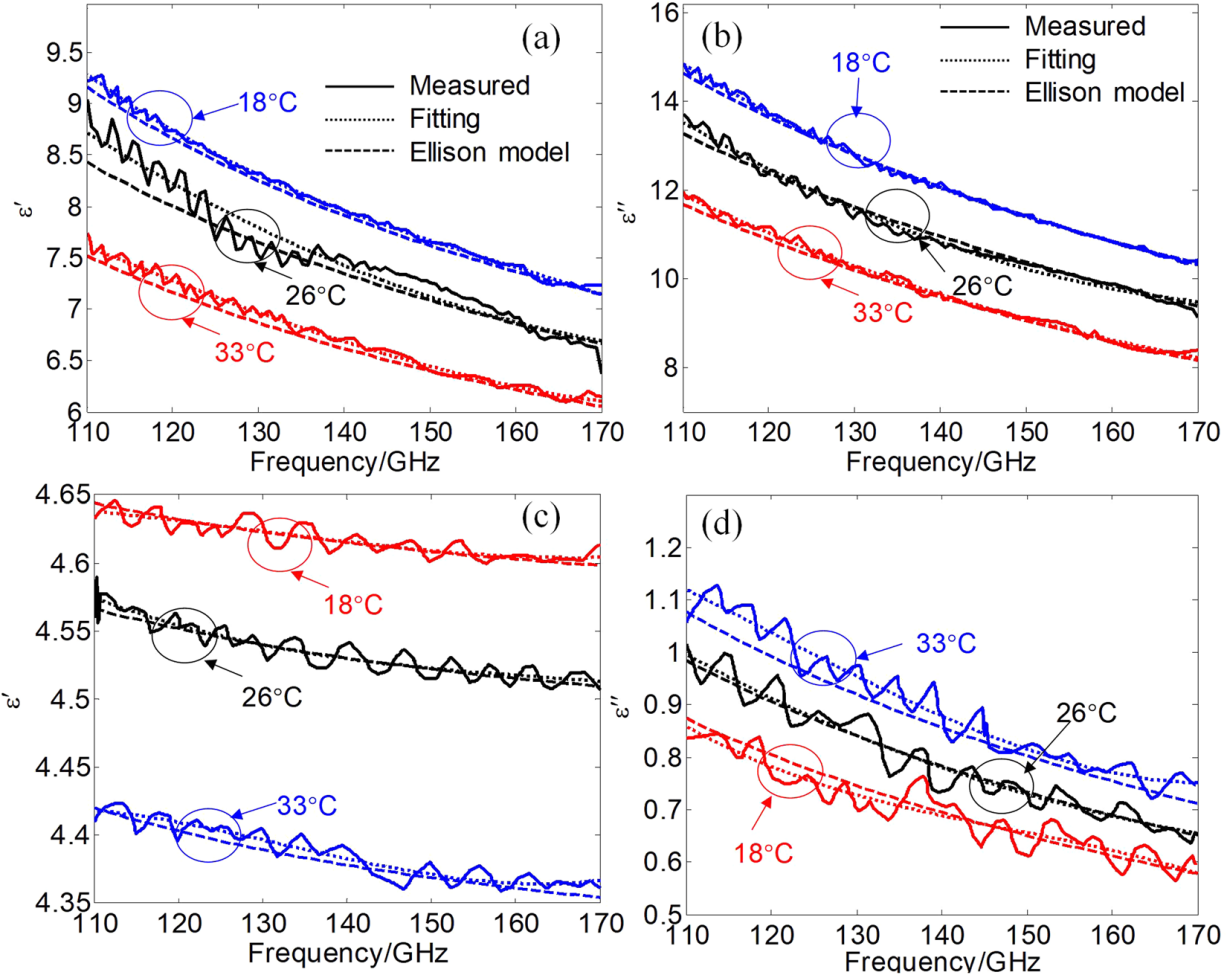
Comparison of measured results with theoretical model. (**a**) Real part of water, (**b**) imaginary part of water, (**c**) real part of methanol, (**d**) imaginary part of methanol.

The retrieved permittivity for the DI-water was plotted in Fig. 8. The solid lines are retrieved data. A three-order polynomial fitting was applied to smooth off the ripples, and the fitting results were plotted in dotted lines. To verify the accuracy of the QO measurement, the Ellison model^10^ for water and the Jordan model^27^ for methanol were also plotted, as shown in dashed lines. It has to be mentioned that the Jordan model only presents data at 10 °C, 20 °C, 30 °C, and 40 °C. The model here was constructed by using a linear extrapolation from the original data.

The relative error to the Ellison model can be estimated at 112 GHz, where both the real and imaginary parts show largest discrepancy. The raw data show a largest discrepancy of 4.0% for the real part and 5.9% for the imaginary part. The fitting values of the real and imaginary parts are 8.72 and 13.54, respectively. And the Ellison model predicts the real and imaginary parts to be 8.43 and 13.28, respectively. It can be calculated that the relative errors for the real and imaginary parts are 3.5% and 2.0%, respectively. The maximal error for the methanol is 3.9% at 110 GHz. Therefore, the relative error can be estimated to be within 4%.

The values of the permittivity of water at a few frequencies are extrapolated from the fitting lines, and are listed in Tables 1 and 2, for water and methanol, respectively. The deviation of the raw data from the fitting data is also presented as $\Delta {\varepsilon ^{\prime} }_{{\rm{r}}}$ and $\Delta {\varepsilon ^{\prime\prime} }_{{\rm{r}}}$. It is seen in Table 1 that both the real and imaginary parts decrease with the increasing frequency. Also, it is shown that the imaginary part is larger than the real part, which explains why electromagnetic waves suffer significant loss in the mm wave range since water is very absorptive in this range. With the increase in temperature, both parts decrease accordingly. For methanol, both parts decrease with frequency, but are less significant than that of water do. It is also found that the real part decreases with increase in temperature, while the imaginary part increases with the increase in temperature.

**Table 1:** The permittivity of water in the D-band, ${\varepsilon ^{\prime} }_{{\rm{r}}}$ and ${\varepsilon ^{\prime\prime} }_{{\rm{r}}}$ are the fitting values, $\Delta {\varepsilon ^{\prime} }_{{\rm{r}}}$ and $\Delta {\varepsilon ^{\prime\prime} }_{{\rm{r}}}$ represent the deviation of the raw data from the fitting data.

Freq (GHz)		110	115	120	125	130	135	140	145	150	155	160	165	170
${\varepsilon ^{\prime} }_{{\rm{r}}}$	18 °C	9.29	9.00	8.74	8.51	8.31	8.12	7.96	7.81	7.67	7.54	7.41	7.28	7.14
	26 °C	8.72	8.46	8.22	8.00	7.79	7.60	7.42	7.26	7.12	6.99	6.88	6.78	6.70
	33 °C	7.61	7.44	7.28	7.12	6.96	6.82	6.69	6.56	6.44	6.34	6.25	6.17	6.11
${\varepsilon ^{\prime\prime} }_{{\rm{r}}}$	18 °C	14.86	14.27	13.74	13.25	12.83	12.43	12.05	11.72	11.40	11.12	10.85	10.58	10.33
	26 °C	13.54	12.99	12.48	12.02	11.57	11.19	10.82	10.50	10.22	9.99	9.78	9.61	9.49
	33 °C	11.9	11.45	11.04	10.65	10.3	9.95	9.64	9.37	9.09	8.85	8.63	8.42	8.22
$\Delta {\varepsilon ^{\prime} }_{{\rm{r}}}$	18 °C	−0.07	0.06	−0.10	−0.01	0.01	−0.01	0.00	0.03	−0.01	0.00	0.02	−0.04	0.10
	26 °C	0.32	0.02	−0.02	−0.33	−0.25	−0.17	0.09	0.14	0.13	0.11	0.04	−0.07	−0.33
	33 °C	0.13	0.00	0.01	−0.04	0.01	−0.04	0.02	0.05	−0.04	0.01	0.01	−0.06	0.04
$\Delta {\varepsilon ^{\prime\prime} }_{{\rm{r}}}$	18 °C	0.00	0.09	−0.03	0.12	−0.05	−0.06	0.11	0.05	0.04	0.00	0.00	−0.03	0.09
	26 °C	0.17	0.25	−0.04	−0.10	−0.06	−0.20	−0.01	0.04	0.13	0.11	0.10	−0.10	−0.35
	33 °C	0.07	0.02	0.15	−0.06	−0.05	0.10	0.05	−0.04	0.04	0.10	−0.06	−0.08	0.17

**Table 2:** The permittivity of methanol in the D-band, ${\varepsilon ^{\prime} }_{{\rm{r}}}$ and ${\varepsilon ^{\prime\prime} }_{{\rm{r}}}$ are the fitting values, $\Delta {\varepsilon ^{\prime} }_{{\rm{r}}}$ and $\Delta {\varepsilon ^{\prime\prime} }_{{\rm{r}}}$ represent the deviation of the raw data from the fitting data.

Freq (GHz)		110	115	120	125	130	135	140	145	150	155	160	165	170
${\varepsilon ^{\prime} }_{{\rm{r}}}$	18°C	4.638	4.635	4.631	4.627	4.624	4.620	4.617	4.612	4.609	4.607	4.605	4.604	4.604
	26°C	4.575	4.564	4.554	4.547	4.540	4.535	4.530	4.526	4.523	4.520	4.518	4.515	4.513
	33°C	4.419	4.415	4.410	4.403	4.397	4.390	4.383	4.376	4.371	4.367	4.365	4.365	4.366
${\varepsilon ^{\prime\prime} }_{{\rm{r}}}$	18°C	0.860	0.818	0.782	0.753	0.728	0.707	0.689	0.672	0.656	0.640	0.623	0.603	0.579
	26°C	0.997	0.954	0.913	0.876	0.841	0.809	0.780	0.754	0.729	0.708	0.688	0.671	0.655
	33°C	1.120	1.079	1.037	0.996	0.955	0.915	0.878	0.845	0.821	0.791	0.771	0.758	0.751
$\Delta {\varepsilon ^{\prime} }_{{\rm{r}}}$	18°C	−0.006	−0.006	−0.004	−0.006	0.004	0.010	0.000	0.000	−0.007	−0.002	0.001	−0.001	0.009
	26°C	−0.015	0.003	0.000	0.006	−0.003	−0.001	0.011	−0.004	−0.007	−0.007	−0.004	−0.005	−0.006
	33°C	−0.010	−0.013	−0.014	0.003	0.008	0.008	0.007	−0.010	0.008	0.008	0.002	−0.001	−0.005
$\Delta {\varepsilon ^{\prime\prime} }_{{\rm{r}}}$	18°C	−0.024	0.006	−0.017	0.003	−0.025	0.002	0.020	−0.030	−0.025	0.010	−0.024	0.016	0.015
	26°C	0.019	0.041	0.030	0.005	0.034	−0.035	−0.025	−0.018	0.001	0.017	0.005	−0.012	0.004
	33°C	−0.042	−0.002	0.005	−0.021	0.019	0.017	0.004	−0.003	0.001	0.014	0.006	−0.034	−0.003

## Discussion

In a FS system, solid samples can be measured using a freestanding plate. In contrast, liquid samples have to be measured in a properly designed sample holder. Unlike solid samples, many factors contribute to the measurement error, such as temperature measurement accuracy, the thickness of the gap between the two glass plates, the stability of the electronic system.

As can been seen from the measured results, the permittivities of water and methanol have very strong temperature dependence. For instance, the real part of water in the D-band has a temperature variation about 0.11 per centigrade, leading to a relative error around 1.2%. And the imaginary has a temperature variation about 0.14–0.2, leading to a relative error of 1.4–1.5%.

For methanol, the relative error due to unit centigrade temperature variation is about 0.3%. Temperature measurement of high accuracy is an efficient way to minimize measurement error. The temperature measurement error in this work is within 1 centigrade. Now, temperature sensors with accuracy as good as 0.1 centigrade are commercially available, such as *FOB* series from *OMEGA* Ltd. Therefore, the temperature caused relative error can be minimized to 0.2%.

The thickness of the gap is also an influential factor. By calculation using the flow-chart method, a measurement error of 20 μm in thickness leads to a relative error of 1.0–1.5%. This is the case in this work. Currently, length measurement based on laser techniques can be as high as 1μm. The measurement error due to thickness can be technically reduced to 0.1%.

The stability of electronic systems can be now maintained within 0.1 dB in amplitude and 1° in phase, which would maximumly cause a relative error of 1.0%. Warming up the electronic system for half an hour prior to measurement would be much beneficiary.

Other factors such as the cross polarization of the system may also be influential to the measurement, but it only affects anisotropic samples since isotropic sample does not change the state of polarization of the incident wave.

## Conclusions

A quasi-optical free space spectroscopy has been applied for dielectric measurement in the millimeter wave range. The signal-flow graph method has been established for multi-layer structure analysis. The permittivity of de-ionized water and methanol have been characterized. Compared to the theoretical models, it is demonstrated that this spectroscopy has an accuracy within 4% in the D-band. It is also shown that water is a high-loss medium in the D-band.

## Supplementary information

supplementary Material - Signal flow chart for tri-layer structure
