# Supplementary material for: Characterization of the dielectric properties of water and methanol in the D-band using a quasi-optical spectroscopy
Source: Sci Rep. 2019 Dec 6;9:18562. doi: 10.1038/s41598-019-55126-6 (PMC6898198; doi:10.1038/s41598-019-55126-6)
Supplement: Supplementary file 1 — supplementary Material - Signal flow chart for tri-layer structure [file 41598_2019_55126_MOESM1_ESM.pdf]

Characterization of the dielectric properties of water and methanol in the D-band using a quasi-optical spectroscopy

Xiaoming Liu[1,2,*], Junsheng Yu[3]

[1]School of Physics and Electronic Information, Anhui Normal University, Wuhu Anhui, 241002, China, Anhui provincial engineering laboratory on information fusion and control of intelligent robot

[2]School of Electronic Engineering and Computer Science, Queen Mary University of London, London, E1 4NS, UK

[3]School of Electronic Engineering, Beijing University of Posts and Telecommunications, Beijing, 100876, China

*Correspondence to [xiaoming.liu@ahnu.edu.cn]

Signal flow char for tri-layer Structure

This document gives a description on how to derive the reflection and transmission coefficients for the tri-layer structure.

Step -1: the first layer from the righthand side

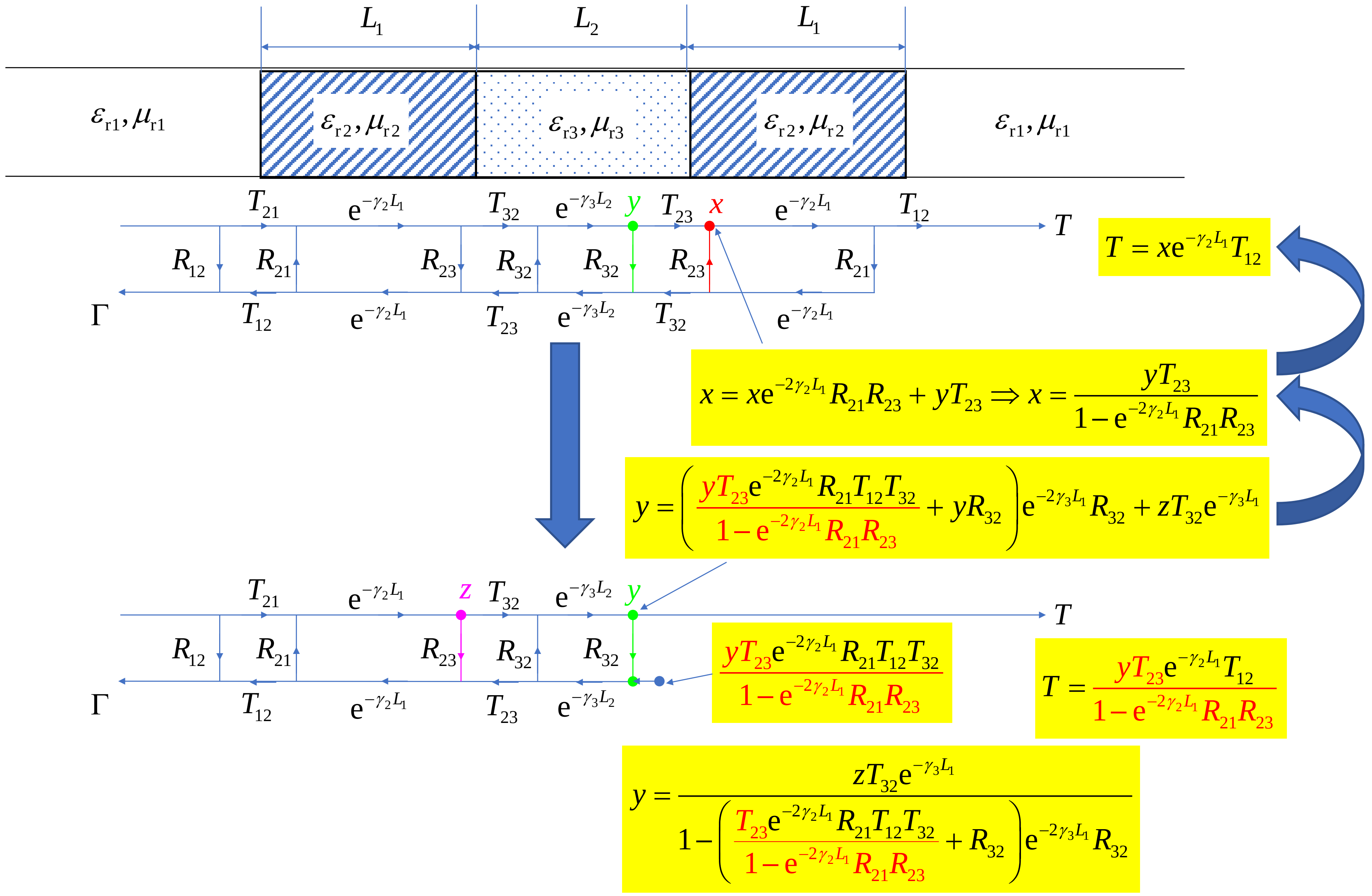

Step – 2: for transmission coefficients (flow chart illustration)

$$y=\frac{zT_{32}e^{-\gamma_3 L_1}}{1-\left(\frac{T_{23}e^{-2\gamma_2 L_1}R_{21}T_{12}T_{32}}{1-e^{-2\gamma_2 L_1}R_{21}R_{23}}+R_{32}\right)e^{-2\gamma_3 L_1}R_{32}}$$

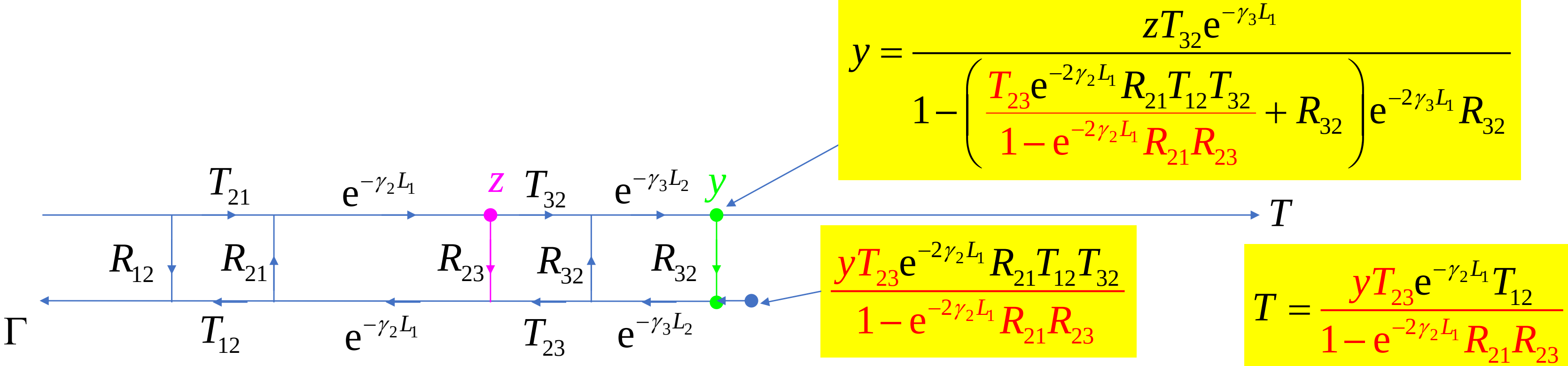

$$\frac{yT_{23}e^{-2\gamma_2 L_1}R_{21}T_{12}T_{32}}{1-e^{-2\gamma_2 L_1}R_{21}R_{23}}$$

$$T=\frac{yT_{23}e^{-\gamma_2 L_1}T_{12}}{1-e^{-2\gamma_2 L_1}R_{21}R_{23}}$$

$$z=T_{21}e^{-\gamma_2 L_1}+e^{-2\gamma_2 L_1}R_{21}\left[zR_{23}+y\left(\frac{T_{23}e^{-2\gamma_2 L_1}R_{21}T_{12}T_{32}}{1-e^{-2\gamma_2 L_1}R_{21}R_{23}}+R_{32}\right)e^{-\gamma_3 L_2}T_{23}\right]$$

$$=T_{21}e^{-\gamma_2 L_1}+ze^{-2\gamma_2 L_1}R_{21}\left[R_{23}+\frac{\left(\frac{T_{23}e^{-2\gamma_2 L_1}R_{21}T_{12}T_{32}}{1-e^{-2\gamma_2 L_1}R_{21}R_{23}}+R_{32}\right)e^{-\gamma_3 L_2}T_{23}T_{32}e^{-\gamma_3 L_1}}{1-\left(\frac{T_{23}e^{-2\gamma_2 L_1}R_{21}T_{12}T_{32}}{1-e^{-2\gamma_2 L_1}R_{21}R_{23}}+R_{32}\right)e^{-2\gamma_3 L_1}R_{32}}\right]$$

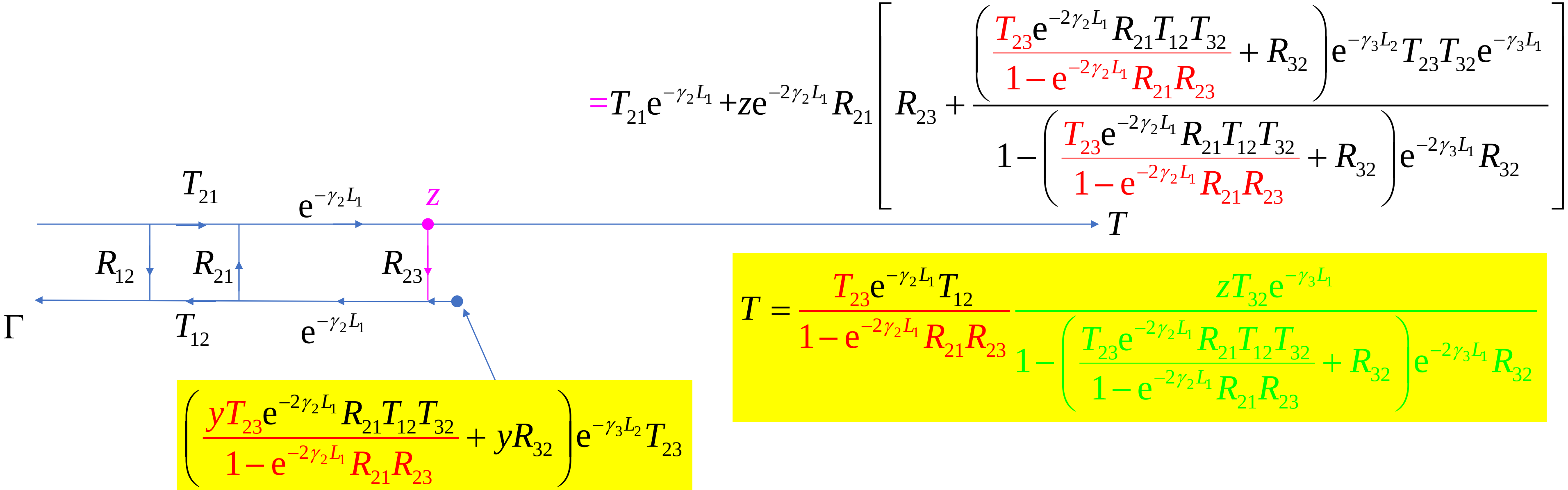

$$T=\frac{T_{23}e^{-\gamma_2 L_1}T_{12}}{1-e^{-2\gamma_2 L_1}R_{21}R_{23}}\cdot\frac{zT_{32}e^{-\gamma_3 L_1}}{1-\left(\frac{T_{23}e^{-2\gamma_2 L_1}R_{21}T_{12}T_{32}}{1-e^{-2\gamma_2 L_1}R_{21}R_{23}}+R_{32}\right)e^{-2\gamma_3 L_1}R_{32}}$$

$$\left(\frac{yT_{23}e^{-2\gamma_2 L_1}R_{21}T_{12}T_{32}}{1-e^{-2\gamma_2 L_1}R_{21}R_{23}}+yR_{32}\right)e^{-\gamma_3 L_2}T_{23}$$

Step – 2: for transmission coefficients (Final expression)

$$z = T_{21} e^{-\gamma_2 L_1} + e^{-2\gamma_2 L_1} R_{21} \left[z R_{23} + y \left(\frac{T_{23} e^{-2\gamma_2 L_1} R_{21} T_{12} T_{32}}{1 - e^{-2\gamma_2 L_1} R_{21} R_{23}} + R_{32} \right) e^{-\gamma_3 L_2} T_{23} \right]$$

$$= T_{21} e^{-\gamma_2 L_1} + z e^{-2\gamma_2 L_1} R_{21} \left[R_{23} + \frac{\left(\frac{T_{23} e^{-2\gamma_2 L_1} R_{21} T_{12} T_{32}}{1 - e^{-2\gamma_2 L_1} R_{21} R_{23}} + R_{32} \right) e^{-\gamma_3 L_2} T_{23} T_{32} e^{-\gamma_3 L_1}}{1 - \left(\frac{T_{23} e^{-2\gamma_2 L_1} R_{21} T_{12} T_{32}}{1 - e^{-2\gamma_2 L_1} R_{21} R_{23}} + R_{32} \right) e^{-2\gamma_3 L_1} R_{32}} \right]$$

$$z = \frac{T_{21} e^{-\gamma_2 L_1}}{1 - e^{-2\gamma_2 L_1} R_{21} \left[R_{23} + \frac{\left(\frac{T_{23} e^{-2\gamma_2 L_1} R_{21} T_{12} T_{32}}{1 - e^{-2\gamma_2 L_1} R_{21} R_{23}} + R_{32} \right) e^{-\gamma_3 L_2} T_{23} T_{32} e^{-\gamma_3 L_1}}{1 - \left(\frac{T_{23} e^{-2\gamma_2 L_1} R_{21} T_{12} T_{32}}{1 - e^{-2\gamma_2 L_1} R_{21} R_{23}} + R_{32} \right) e^{-2\gamma_3 L_1} R_{32}} \right]}$$

$$T = \frac{T_{23} e^{-\gamma_2 L_1} T_{12}}{1 - e^{-2\gamma_2 L_1} R_{21} R_{23}} \frac{T_{32} e^{-\gamma_3 L_1}}{1 - \left(\frac{T_{23} e^{-2\gamma_2 L_1} R_{21} T_{12} T_{32}}{1 - e^{-2\gamma_2 L_1} R_{21} R_{23}} + R_{32} \right) e^{-2\gamma_3 L_1} R_{32}} \frac{T_{21} e^{-\gamma_2 L_1}}{1 - e^{-2\gamma_2 L_1} R_{21} \left[R_{23} + \frac{\left(\frac{T_{23} e^{-2\gamma_2 L_1} R_{21} T_{12} T_{32}}{1 - e^{-2\gamma_2 L_1} R_{21} R_{23}} + R_{32} \right) e^{-\gamma_3 L_2} T_{23} T_{32} e^{-\gamma_3 L_1}}{1 - \left(\frac{T_{23} e^{-2\gamma_2 L_1} R_{21} T_{12} T_{32}}{1 - e^{-2\gamma_2 L_1} R_{21} R_{23}} + R_{32} \right) e^{-2\gamma_3 L_1} R_{32}} \right]}$$

Step – 3: for reflection coefficients (flow chart illustration)

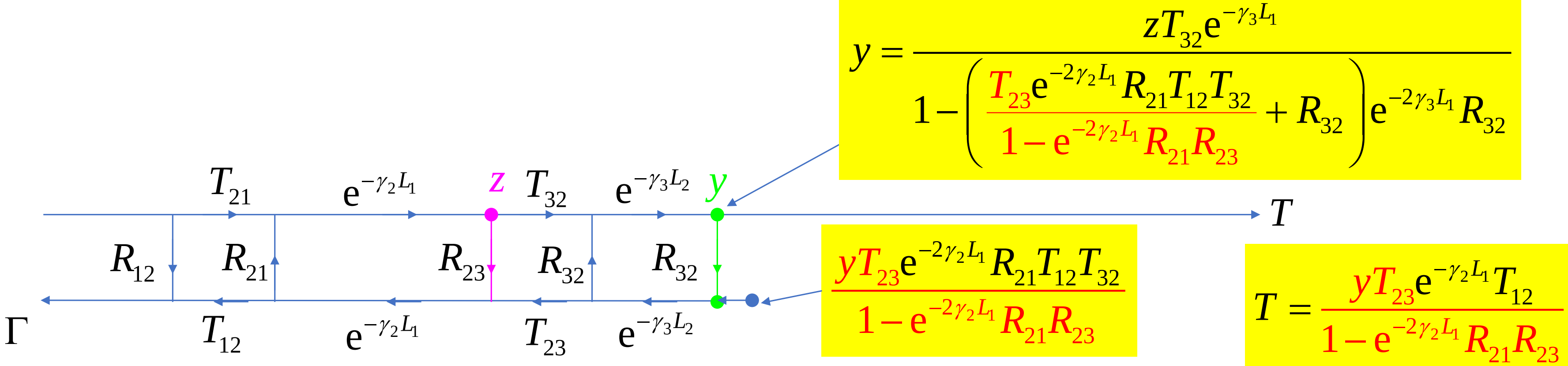

$$y = \frac{zT_{32}e^{-\gamma_3 L_1}}{1-\left(\frac{T_{23}e^{-2\gamma_2 L_1}R_{21}T_{12}T_{32}}{1-e^{-2\gamma_2 L_1}R_{21}R_{23}}+R_{32}\right)e^{-2\gamma_3 L_1}R_{32}}$$

$$\frac{yT_{23}e^{-2\gamma_2 L_1}R_{21}T_{12}T_{32}}{1-e^{-2\gamma_2 L_1}R_{21}R_{23}}$$

$$T = \frac{yT_{23}e^{-\gamma_2 L_1}T_{12}}{1-e^{-2\gamma_2 L_1}R_{21}R_{23}}$$

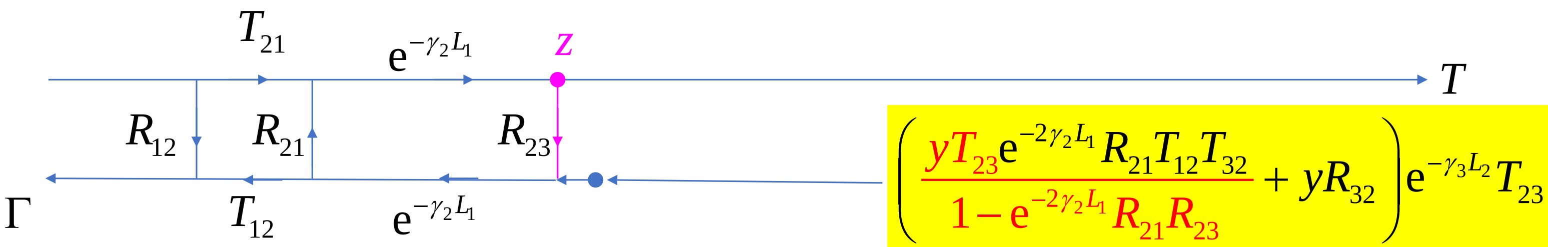

$$\left(\frac{yT_{23}e^{-2\gamma_2 L_1}R_{21}T_{12}T_{32}}{1-e^{-2\gamma_2 L_1}R_{21}R_{23}}+yR_{32}\right)e^{-\gamma_3 L_2}T_{23}$$

$$\Gamma = R_{12}+e^{-2\gamma_2 L_1}T_{12}\left[zR_{23}+y\left(\frac{T_{23}e^{-2\gamma_2 L_1}R_{21}T_{12}T_{32}}{1-e^{-2\gamma_2 L_1}R_{21}R_{23}}+R_{32}\right)e^{-\gamma_3 L_2}T_{23}\right]$$

$$= R_{12}+ze^{-2\gamma_2 L_1}T_{12}\left[R_{23}+\frac{T_{32}e^{-\gamma_3 L_1}}{1-\left(\frac{T_{23}e^{-2\gamma_2 L_1}R_{21}T_{12}T_{32}}{1-e^{-2\gamma_2 L_1}R_{21}R_{23}}+R_{32}\right)e^{-2\gamma_3 L_1}R_{32}}\left(\frac{T_{23}e^{-2\gamma_2 L_1}R_{21}T_{12}T_{32}}{1-e^{-2\gamma_2 L_1}R_{21}R_{23}}+R_{32}\right)e^{-\gamma_3 L_2}T_{23}\right]$$

Step – 3: for reflection coefficients (Final expression)

$$\Gamma = R_{12} + e^{-2\gamma_2 L_1} T_{12}\left[zR_{23} + y\left(\frac{T_{23} e^{-2\gamma_2 L_1} R_{21} T_{12} T_{32}}{1 - e^{-2\gamma_2 L_1} R_{21} R_{23}} + R_{32} \right) e^{-\gamma_3 L_2} T_{23} \right]$$

$$= R_{12} + z e^{-2\gamma_2 L_1} T_{12}\left[R_{23} + \frac{T_{32} e^{-\gamma_3 L_1}}{1 - \left(\frac{T_{23} e^{-2\gamma_2 L_1} R_{21} T_{12} T_{32}}{1 - e^{-2\gamma_2 L_1} R_{21} R_{23}} + R_{32} \right) e^{-2\gamma_3 L_1} R_{32}} \left(\frac{T_{23} e^{-2\gamma_2 L_1} R_{21} T_{12} T_{32}}{1 - e^{-2\gamma_2 L_1} R_{21} R_{23}} + R_{32} \right) e^{-\gamma_3 L_2} T_{23} \right]$$

$$\Gamma = R_{12} + \frac{T_{21} e^{-\gamma_2 L_1} e^{-2\gamma_2 L_1} T_{12}\left[R_{23} + \frac{T_{32} e^{-\gamma_3 L_1}}{1 - \left(\frac{T_{23} e^{-2\gamma_2 L_1} R_{21} T_{12} T_{32}}{1 - e^{-2\gamma_2 L_1} R_{21} R_{23}} + R_{32} \right) e^{-2\gamma_3 L_1} R_{32}} \left(\frac{T_{23} e^{-2\gamma_2 L_1} R_{21} T_{12} T_{32}}{1 - e^{-2\gamma_2 L_1} R_{21} R_{23}} + R_{32} \right) e^{-\gamma_3 L_2} T_{23} \right]}{1 - e^{-2\gamma_2 L_1} R_{21}\left[R_{23} + \frac{\left(\frac{T_{23} e^{-2\gamma_2 L_1} R_{21} T_{12} T_{32}}{1 - e^{-2\gamma_2 L_1} R_{21} R_{23}} + R_{32} \right) e^{-\gamma_3 L_2} T_{23} T_{32} e^{-\gamma_3 L_1}}{1 - \left(\frac{T_{23} e^{-2\gamma_2 L_1} R_{21} T_{12} T_{32}}{1 - e^{-2\gamma_2 L_1} R_{21} R_{23}} + R_{32} \right) e^{-2\gamma_3 L_1} R_{32}} \right]}$$
